# Association between dietary energy density with mental health and sleep quality in women with overweight/obesity

**DOI:** 10.1186/s13104-020-05025-1

**Published:** 2020-03-30

**Authors:** Niloofar Sadat Maddahi, Habib Yarizadeh, Leila Setayesh, Yasaman Nasir, Shahab Alizadeh, Khadijeh Mirzaei

**Affiliations:** Department of Community Nutrition, School of Nutritional Sciences and Dietetics, Tehran University of Medical Sciences (TUMS), P.O. Box: 14155-6117, Tehran, Iran

**Keywords:** Dietary energy density, Mental health, Stress, Depression, Anxiety, Sleep quality

## Abstract

**Objective:**

Mental health, sleep quality, and dietary intake are interlinked. Impairment of mental health and low sleep quality may contribute to obesity through the consumption of diets high in energy density. Nevertheless, it is not clear whether dietary energy density (DED) influences mental health. This study aimed to examine the association of DED with mental health indices, including depression, anxiety, stress, and sleep quality in women with overweight/obesity.

**Results:**

There was a decreasing trajectory in serum triglyceride across quartiles of DED (from Q1 to Q4) in the crude analysis and also after adjustment for age, BMI, and physical activity After adjustment for age, BMI, and physical activity, subjects in the highest quartile of DED had higher systolic and diastolic blood pressure. DED was significantly associated with increased odds of stress in the crude (OR = 2.15, 95% CI 1.01–4.56, p = 0.04) and adjusted model for age, BMI, and physical activity (OR = 2.56, 95% CI 1.13–5.79, p = 0.02). No significant relationship was observed between DED and depression, anxiety and sleep quality. In conclusion, current study shows preliminary evidence of an association between DED and stress.

## Introduction

The prevalence of obesity has progressively risen in all parts of the world during the last decades and has become a main public health concern. It is well-known that the positive energy balance usually resulted from excessive intake of energy, is the fundamental dietary factor associated with weight gain [1]. Assessment of the overall impact of diet is commonly preferred for the evaluation of single dietary constituents, such as energy intake. Among diet quality indices, dietary energy density (DED), as a measure of the whole diet, has been at the focus of many recent investigations [2]. DED is a comparatively new dietary index that has an important role in body weight control [3], which is defined as the amount of energy per unit weight of a food or beverage, usually reported as kilocalories/100 g [4]. High-energy dense diets are rich in fat, because dietary fat provides the greatest amount of energy per gram, but are low in vegetable, fruit, and fiber [2, 5, 6]. It has been found that higher DED is associated with the risk of obesity [7] and obesity-related diseases [8], indicating that adopting diets with lower DED are important preventive approaches for obesity-related complications.

Furthermore, obesity is reported to be related to impaired mental health such as anxiety, stress, depression and low quality of sleep [9, 10]; and on the other side, mental health, sleep behavior and, dietary intake are interlinked [11]. There is inadequate evidence in the emerging field of “relationship between dietary intake, mental health, and sleep quality”. The majority of studies in this area of research have concentrated on food items, macronutrients, single nutrients or energy intakes [12, 13]. Nevertheless, limited research with conflicting results [11, 14–18] exists on the association between DED and indices of sleep quality and mental health. There is a higher prevalence of poor sleep quality [19], depression, and anxiety disorders [19, 20] in women than in men. Moreover, women are more often affected by problems with their eating behaviors, such as craving for special foods [21], which results to a higher rate of obesity compared with men [22]. Thus, in the present study, we selected women as the study population. Thus, this study was performed to assed the relation of dietary energy density to mental health and sleep quality in women with overweight and obesity.

## Main text

### Method

#### Study population

A total of 301 women with overweight/obesity took part in the present cross-sectional study. All subjects were randomly recruited from individuals referring to health centers in Tehran during 2016–2017. Inclusion criteria were age 18–56 years, being overweight or obese (body mass index (BMI) ≥ 25), absence of any acute or chronic infection, no alcohol or drug abuse, no history of hypertension, and not being pregnant. Based on exclusion criteria, prospective subjects with a history of cardiovascular disease, cancer, sustained hypertension, diabetes, thyroid disease, cancer, acute or chronic infections, liver and kidney disease, and smokers were excluded from the study. Written informed consent was obtained from all participants before taking part in the study. The study protocol was approved by the local ethical committee of Tehran University of Medical Sciences (IR. TUMS.VCR.REC. 41017-33893).

#### Body composition and anthropometric measurement

The body composition of all participants was mustered using a body composition analyzer (InBody770 scanner; InBody, Seoul, Korea) by following the manufacturer’s protocol. The weight of the individuals was measured with the use of a digital scale (Seca, Hamburg, Germany) in light clothing and without shoes with precision near to 0.1 kg. The height of participants was evaluated by a seca stadiometer, with exactness close to 0.1 cm. BMI was calculated as weight (kg)/hieght^2^ (m). Waist (WC) and hip circumference (HC) were measured in the smallest girth and the largest girth, respectively, with accuracy nearest to 0.1 cm.

#### Evaluation of food intake and DED

To assess the dietary intake of participants, a semi-quantitative food frequency questionnaire (sq-FFQ) with 147 Iranian food items, containing a list of foods with standard serving sizes was used. FFQ assesses the usual food intake over the previous year. The high reliability and validity of the FFQ have been confirmed previously [23]. All FFQ questionnaires were completed by trained nutritionists. The energy of food consumed was evaluated using Nutritionist 4 software. DED was calculated by dividing the total dietary energy intake from consumed food (kcal/d) by the total weight (g/d) of consumed foods (excluding beverages) [24]. The unit for the DED was kcal/g.

#### Assessment of mental health and sleep quality

Mental health was evaluated with the use of the 21-question version of the Depression Anxiety Stress Scales (DASS-21), which evaluates depression, anxiety and, stress over the past week [25]. The Pittsburgh Sleep Quality Index (PSQI) [26] was applied to subjectively measure the sleep quality of participants. It evaluates usual sleep habits during the past month. Total scores could range from 0 to 21, with a global sum of “5” or greater indicates poor sleep quality [26].

#### Measurement of biochemical parameters

Blood samples were obtained in the early morning between 8:00 and 10:00 a.m. after a 10- to 12-h overnight fasting. Serum concentrations of high-density lipoprotein cholesterol (HDL-C), total cholesterol (TC), triglyceride (TG), and low-density lipoprotein cholesterol (LDL-C) were evaluated by using of enzymatic approaches using related kits (Pars Azemun, Iran) and autoanalyzer system. Insulin level was assessed using an ELISA kit (Human insulin ELISA kit, DRG Pharmaceuticals, GmbH, Germany), and fasting concentration of glucose was measured using a glucose oxidase method. Serum high-sensitive C-reactive protein (hs-CRP) was assessed with the use of the immunoturbidimetric assay.

#### Statistical analysis

DED was categorized into quartiles as followings: Q1 (0.61 to 1.00); Q2 (1.01 to 1.18); Q3 (1.19 to 1.44); and Q4 (1.45 to 3.79). Analyses of continuous variables to assess differences among quartile of DED were performed using the one-way analysis of variance (ANOVA). The results were adjusted for multiple comparisons using the tukey’s post hoc test. Analysis of Covariance (ANCOVA) was then used to find the difference between the means of investigated variables across quartiles of DED adjusted for age, physical activity and BMI. The logistic regression analysis was applied to find the relation of DED to sleep quality, stress, anxiety, and depression; this model was then adjusted for age, physical activity, and BMI. The level of significance was set at a probability of ≤ 0.05 for all tests. All statistical analyses were conducted using a statistical Package for Social Science (Version 22.0; SPSS Inc., Chicago IL, USA).

### Results

A total of 301 women, aged 18–56 year, participated in this study and 293 subjects completed measurements. General characteristics of the study subjects are reported in Table 1. The mean age and BMI of participants were 36.39 ± 8.41 and 30.77 ± 3.79, respectively. There was a decreasing trajectory in serum TG across quartiles of DED (from Q1 to Q4) in the crude analysis and also after adjustment for age, BMI, and physical activity (p ≤ 0.05). Moreover, after adjustment for age, BMI, and physical activity, subjects in the highest quartile of DED had higher systolic (p = 0.01) and diastolic blood pressure (p = 0.04) than those in the lowest quartile. No significant differences were observed in depression, anxiety, stress, and sleep quality across the categories of DED (Table 2).

**Table 1:** Characteristics of the study participants

Variable	Mean ± SD	Minimum	Maximum
Age (years)	36.39 ± 8.41	18.00	56.00
Body weight (Kg)	80.22 ± 11.28	59.50	122.40
BMI (Kg/m^2^)	30.77 ± 3.79	25.00	40.70
Total cholesterol (mg/dL)	183.78 ± 35.33	104.00	344.00
TG (mg/dL)	120.78 ± 68.69	37.00	512.00
HDL (mg/dL)	46.36 ± 10.64	18.00	82.00
LDL (mg/dL)	94.41 ± 24.05	34.00	156.00
SBP (mmHg)	111.24 ± 13.41	76.00	159.00
DBP (mmHg)	77.69 ± 9.61	51.00	111.00
hs-CRP (mg/L)	4.25 ± 4.59	00.00	22.73
Insulin (μIU/mL)	15.59 ± 6.02	6.67	65.89
HOMA	3.40 ± 1.52	1.29	16.59
FBS (mmol/L)	4.85 ± 0.53	3.72	7.61
BFM (Kg)	33.52 ± 7.74	19.40	59.40
FFM (Kg)	46.68 ± 5.50	35.30	67.70
WC (cm)	98.56 ± 9.43	80.10	131.30
Depression	5.23 ± 4.69	00.00	20.00
Anxiety	5.08 ± 3.98	00.00	19.00
Stress	7.87 ± 5.02	00.00	21.00
PSQI	5.65 ± 3.44	00.00	21.00
DED	1.23 ± 0.34	0.61	3.79

*SD* Standard deviation, *BMI* body mass index, *TG* triglyceride, *LDL* low density lipoprotein, *HDL* high density lipoprotein, *FBS* fasting blood sugar, *SBP* systolic blood pressure, *DBP* diastolic blood pressure, *BFM* body fat mas*s*, *FFM* fat free mass, *hs*-*CRP* high-sensitivity C-reactive protein, *HOMA*-*IR* homeostatic model assessment of insulin resistance

**Table 2:** Mean and SD of anthropometric body composition, blood parameters and blood pressure across the quartiles of DED

Variable	Q1	Q2	Q3	Q4	P Q2 vs. Q1	P Q3 vs. Q1	P Q4 vs. Q1	P Q3 vs. Q2	P Q4 vs. Q2	P Q4 vs. Q3	$$ \begin{array}{*{20}c} {ANOVA } \\ {p\_value} \\ \end{array}_{\alpha } $$	$$ \begin{array}{*{20}c} {ANOVA } \\ {p\_value} \\ \end{array}_{\beta } $$
Age (years)	38.20 ± 7.58	35.90 ± 8.66	35.73 ± 9.27	36.16 ± 8.41	0.38	0.32	0.49	1.00	0.99	0.99	0.28	0.25^a^
Body weight (Kg)	79.46 ± 11.24	80.62 ± 11.28	81.07 ± 10.71	78.88 ± 10.89	0.92	0.82	0.99	0.99	0.88	0.64	0.62	0.74^b^
BMI (Kg/m^2^)	30.48 ± 3.46	30.95 ± 3.96	30.88 ± 3.67	30.76 ± 3.94	0.88	0.92	0.97	1.00	0.99	0.99	0.89	0.92^b^
Total cholesterol (mg/dL)	183.85 ± 32.57	184.91 ± 37.12	182.92 ± 34.89	180.98 ± 38.93	0.99	0.99	0.97	0.99	0.93	0.99	0.94	0.87^c^
TG (mg/dL)	117.04 ± 58.96	115.82 ± 67.28	147.03 ± 94.78	103.17 ± 44.16	1.00	0.08	0.68	0.07	0.75	*0.004*	*0.006*	*0.001*^*c*^
HDL (mg/dL)	46.04 ± 10.28	47.03 ± 12.56	45.89 ± 10.15	47.44 ± 9.50	0.95	1.00	0.89	0.94	0.99	0.86	0.83	0.50^c^
LDL (mg/dL)	93.70 ± 24.23	93.91 ± 24.54	97.33 ± 23.09	91.08 ± 24.56	1.00	0.84	0.93	0.87	0.92	0.50	0.58	0.48^c^
SBP (mmHg)	109.35 ± 14.35	111.14 ± 13.67	109.73 ± 12.49	114.72 ± 13.25	0.86	0.99	0.10	0.92	0.42	0.14	0.09	*0.01*^*c*^
DBP (mmHg)	76.17 ± 9.25	76.89 ± 9.07	77.31 ± 9.75	80.12 ± 10.30	0.97	0.90	0.08	0.99	0.21	0.33	0.09	*0.04*^*c*^
hs-CRP (mg/L)	4.12 ± 4.92	4.39 ± 4.50	3.63 ± 4.06	4.74 ± 5.01	0.99	0.93	0.88	0.81	0.97	0.57	0.62	0.55^c^
Insulin (μIU/mL)	14.69 ± 4.97	15.02 ± 4.97	16.76 ± 6.28	15.62 ± 7.77	0.99	0.24	0.83	0.39	0.94	0.73	0.26	0.44^c^
HOMA-IR	3.17 ± 1.25	3.35 ± 1.30	3.57 ± 1.48	3.45 ± 2.03	0.92	0.50	0.77	0.87	0.98	0.97	0.56	0.71^c^
FBS (mmol/L)	4.83 ± 0.52	4.88 ± 0.52	4.80 ± 0.52	4.89 ± 0.58	0.97	0.97	0.96	0.86	1.00	0.83	0.79	0.63^c^
BFM (Kg)	32.65 ± 7.47	33.88 ± 7.79	33.81 ± 7.51	33.48 ± 8.12	0.78	0.81	0.92	1.00	0.99	0.99	0.77	0.94^b^
FFM (Kg)	46.62 ± 5.58	47.04 ± 5.40	47.02 ± 5.70	45.83 ± 5.10	0.96	0.97	0.83	1.00	0.55	0.57	0.52	0.61^b^
WC (cm)	97.60 ± 9.72	99.14 ± 9.74	99.10 ± 8.91	98.05 ± 9.41	0.77	0.78	0.99	1.00	0.90	0.91	0.71	0.82^b^
Depression	4.84 ± 4.77	5.35 ± 4.83	5.57 ± 4.80	4.94 ± 4.30	0.92	0.80	0.99	0.99	0.95	0.86	0.78	0.73^c^
Anxiety	5.40 ± 4.17	5.25 ± 3.75	5.07 ± 3.87	4.31 ± 3.96	0.99	0.96	0.38	0.99	0.51	0.68	0.38	0.55^c^
Stress	7.40 ± 4.76	8.22 ± 5.24	7.92 ± 5.11	7.86 ± 5.06	0.78	0.93	0.95	0.98	0.99	1.00	0.82	0.72^c^
PSQI	6.09 ± 3.87	5.41 ± 3.21	5.62 ± 3.32	5.31 ± 3.08	0.72	0.88	0.66	0.98	0.99	0.96	0.66	0.65^c^

*SD* Standard deviation, *BMI* body mass index, *TG* triglyceride, *LDL* low density lipoprotein, *HDL* high density lipoprotein, *FBS* fasting blood sugar, *SBP* systolic blood pressure, *DBP* diastolic blood pressure, *BFM* body fat mass, *FFM* fat free mass, *hs*-*CRP* high-sensitivity C-reactive protein, *HOMA*-*IR* homeostatic model assessment of insulin resistance

*β* Adjusted model. ^a^adjusted for BMI and physical activity, ^b^adjusted for age and physical activity, ^c^adjusted for age BMI and physical activity

Compared with the lowest quartile, the highest quartile of DED was associated with a 2.15-fold increased odds of stress (OR = 2.15, 95% CI 1.01–4.56, p = 0.04), and even after adjusting for age, BMI, and physical activity this association remained statistically significant (OR = 2.56, 95% CI 1.13–5.79, p = 0.02). No significant association was found between DED and depression, anxiety and sleep quality (Table 3).

**Table 3:** Logistic regression analysis for the association of DED with PSQI, depression, anxiety, and stress

	OR	P
PSQI		
Model 1	0.50 (0.20 to 1.25)	0.14
Model 2	0.52 (0.20 to 1.33)	0.17
Depression		
Model 1	0.88 (0.40 to 1.94)	0.76
Model 2	0.98 (0.44 to 2.18)	0.96
Anxiety		
Model 1	0.53 (0.21 to 1.32)	0.17
Model 2	0.59 (0.22 to 1.52)	0.27
Stress		
Model 1	2.15 (1.01 to 4.56)	*0.04*
Model 2	2.56 (1.13 to 5.79)	*0.02*

Model 1, crude mode l; model 2, adjusted for age, BMI and physical activity

### Discussion

In this study, after adjustment for potential covariates, higher DED was significantly related to 2.56-fold increased odds of stress. Although, no significant relationship was found between DED with anxiety, depression and sleep quality.

In line with our findings, Heath et al. [27], in a study on shift working nurses [N = 52; 46 female; age: 39.8 ± 12.4 years], found that higher levels of stress are associated with higher energy intake. Under stressful conditions, humans desire palatable foods that are energy-dense [28], especially elevated eating of high-sugar, high-fat foods and processed foods [29–32] and a decrease in consumption of main meals, fruits, and vegetables [30, 32, 33]. Moreover, in women, aged 18 to 45 years, with a BMI < 40 kg/m^2^, chronic stress was reported to be related to empty calories (added solid fat and sugar) consumption and evening intake of added sugars [34]. The preference of highly palatable, energy-dense food items is attributed to hormones secreted in stress response, such as cortisol [35]. Behaviorally, during stress people have less energy and time to devote to the preparation of foods; thus, they have an elevated dependence on pre-processed convenience food items, which are frequently rich in energy [36, 37]. The ‘comfort food hypothesis’ proposes that chronic stress could endorse a coping strategy resulting in preference towards food comprising more carbohydrates and saturated fats [33], which have higher energy density.

The western, high fat-high sugar and sweet dietary patterns have been reported to be related to higher odds for depression [38–41]; though, some studies did not reveal such a relationship [42]. Grossniklaus et al. [43], in a study on 87 overweight, working adults; with mean age 41.3 ± 10.2 years; BMI 32.1 ± 6.1 kg/m^2^; 73.6% women, reported that increased depressive symptoms predicted increased food and beverage energy density. There is only one study exploring the relation of DED to depression (Australian men, aged 35–80 years), which consistent with our study, found no association between DED and depression [44]. For anxiety, prior studies found that the saturated fat and added sugars dietary pattern is significantly related to higher anxiety, but in agreement with our results, no association between anxiety level and energy intake was detected in Greek older adults (> 50 years) men and women [45]. Other recent investigations showed that diets high in sugars and fats are associated with higher anxiety levels via changes of protein, glucose, and energy homeostasis, and increases in corticosterone and inflammatory cytokines [46, 47].

Supporting our finding, a study on diabetic nephropathy patients found no association between DED and sleep duration in women and men [15]. However, the majority of studies, such as the study by Stelmach-Mardas (on both genders with mean age 35.6 ± 13.3) [11] and Kjeldsen et al. (on school children) [16] have identified that because of the elevated feeling of appetite, people with low sleep quality have a higher DED or energy intake and preferred food items with high content of carbohydrates and fats, compared with individuals with adequate sleep duration [11, 16]. Mechanistically, sleep modulates the pattern of secretion of two key hormones involved in appetite and energy regulation: ghrelin and leptin [48]. Specifically, partial sleep deprivation appears to lead to increased serum ghrelin and reduced serum leptin, both of which result in elevated appetite [49]. Reliance on the subjective measurement of sleep quality and DED, over varying periods, is a limitation, which may partly explain our inconsistent finding in regards to the possible association between DED and sleep quality. Moreover, previous studies have applied various tools to estimate dietary intake and sleep quality, which the varied methodology might have resulted in inconsistent findings.

### Conclusion

In conclusion, the current study shows preliminary evidence of an association between DED DED and stress. Additional large well-designed studies should be conducted to confirm our findings.

## Limitation

Some limitations of the current study should be considered. Briefly, because of the cross-sectional design of this study, causal inferences could not be extracted; we were not able to declare whether DED was the reason for stress or stress leads to higher DED. There may be some factors, such as socioeconomic or education level of subjects, affecting the DED and mental health that were not measured in the present study. Demographic and mental health data were self-reported, and these data might be subject to social desirability bias and under or over-reporting. As another limitation, the assessment of DED with the use of FFQ is recognized to have potential recall bias, compared with 3-day food records; nevertheless, FFQ is more reliable for estimating long term food intake. Finally, the study population was restricted to women, which limits the generalizability of findings to men; therefore, replication of our results with the use of larger samples in both sexes is essential.

## Data Availability

Data can be reached by contacting the corresponding author.

## Abbreviations

DED: Dietary energy density
BMI: Body mass index
WC: Waist circumference
HC: Hip circumference
FFQ: Food frequency questionnaire
DASS-21: 21-Question version of the Depression Anxiety Stress Scales
PSQI: The Pittsburgh Sleep Quality Index
HDL-C: High-density lipoprotein cholesterol
TC: Cholesterol
TG: Triglyceride
LDL-C: Low-density lipoprotein cholesterol
hs-CRP: Serum high-sensitive C-reactive protein
ANOVA: One-way analysis of variance
ANCOVA: Analysis of Covariance
